# Development of a novel immune infiltration-related diagnostic model for Alzheimer’s disease using bioinformatic strategies

**DOI:** 10.3389/fimmu.2023.1147501

**Published:** 2023-07-20

**Authors:** Xianbo Zhuang, Guifeng Zhang, Mengxin Bao, Guisheng Jiang, Huiting Wang, Shanshan Li, Zheng Wang, Xiujuan Sun

**Affiliations:** ^1^ Department of Neurology, Liaocheng People’s Hospital and Liaocheng Hospital Affiliated to Shandong First Medical University, Liaocheng, China; ^2^ Clinical Laboratory, Liaocheng Veterans Hospital, Liaocheng, China; ^3^ Department of Neurosurgery, Liaocheng Traditional Chinese Medicine Hospital, Liaocheng, China

**Keywords:** Alzheimer’s disease, immune infiltration, WGCNA, machine learning, diagnosis

## Abstract

**Background:**

The pathogenesis of Alzheimer’s disease (AD) is complex and multi-factorial. Increasing evidence has shown the important role of immune infiltration in AD. Thus the current study was designed to identify immune infiltration-related genes and to explore their diagnostic value in AD.

**Methods:**

The expression data of AD patients were downloaded from the GEO database. The limma R package identified differentially expressed genes (DEGs) between AD and controls. The CIBERSORT algorithm identified differentially infiltrated immune cells (DIICs) between AD and controls. DIIC-correlated DEGs were obtained by Pearson correlation analysis. WGCNA was employed to identify DIIC-related modules. Next, LASSO, RFE, and RF machine learning methods were applied to screen robust DIIC-related gene signatures in AD, followed by the construction and validation of a diagnostic nomogram. Detection of the expression of related genes in the peripheral blood of Alzheimer’s disease and healthy volunteers by RT-PCR. In addition, the CTD database predicted chemicals targeting DIIC-related gene signatures in the treatment of AD.

**Results:**

NK cells, M0 macrophages, activated myeloid dendritic cells, resting mast cells, CD8+ T cells, resting memory CD4+ T cells, gamma delta T cells, and M2 macrophages were differentially infiltrated between AD and controls. Pearson analysis identified a total of 277 DIIC-correlated DEGs between AD and controls. Thereafter, 177 DIIC-related genes were further obtained by WGCNA analysis. By LASSO, RFE and RF algorithms, CMTM2, DDIT4, LDHB, NDUFA1, NDUFB2, NDUFS5, RPL17, RPL21, RPL26 and NDUFAF2 were identified as robust gene signature in AD. The results of RT-PCR detection of peripheral blood samples from Alzheimer’s disease and healthy volunteers showed that the expression trend of ten genes screened was consistent with the detection results; among them, the expression levels of CMTM2, DDIT4, LDHB, NDUFS5, and RPL21 are significantly different among groups. Thus, a diagnostic nomogram based on a DIIC-related signature was constructed and validated. Moreover, candidate chemicals targeting those biomarkers in the treatment of AD, such as 4-hydroxy-2-nonenal, rosiglitazone, and resveratrol, were identified in the CTD database.

**Conclusion:**

For the first time, we identified 10 immune infiltration-related biomarkers in AD, which may be helpful for the diagnosis of AD and provide guidance in the treatment of AD.

## Introduction

Alzheimer’s disease (AD) is an irreversible neurodegenerative disease closely associated with aging (1). In the early stages, patients with the disease present with mild memory difficulties that progress to cognitive impairment and various cognitive dysfunctions that affect several areas of cognition, which seriously diminishes the quality of life (2). The prevalence of AD is increasing annually, and according to recent studies, the incidence of Alzheimer’s disease is expected to double and triple in Europe and worldwide by 2050, respectively (3). Despite many efforts to better understand the pathogenesis of AD, current clinical management and treatment are not very effective Early diagnosis and early intervention to slow down the progression of AD appear to be particularly crucial (4). Thus, further exploration of AD at a genetic level, identification of novel biomarkers, and refinement of the diagnostic model are of great significance to improving the intervention and treatment of AD patients.

In current clinical work, the diagnosis of AD is mainly based on the clinical manifestations of typical cognitive decline, with imaging manifestations such as atrophy of the hippocampus and other structures as an auxiliary diagnosis (5, 6). However, the typical clinical symptoms appear mostly in the middle and late stages of the disease process, which makes the early diagnosis of AD very challenging. Nowadays, with the rapid development of sequencing technology, disease sequencing data has increased dramatically (7). The bioinformatics parsing of sequencing data can identify biomarkers such as genes and targets that influence the development of transcendental development, which is of great value for the early diagnosis of AD (8, 9).

Recent studies have revealed that abnormal activation of neuroimmune cells and inflammatory response play a key role in the progression of AD (10). However, the diagnostic value of immune cell-related genes in AD remains unclear. A comprehensive understanding of the genetic and molecular mechanisms underlying immune cell involvement in AD could pave the way for developing targeted therapeutics and personalized treatment strategies. Furthermore, identifying reliable biomarkers and establishing diagnostic models may enable earlier and more accurate detection of AD, facilitating timely interventions and improving patient outcomes. The research team aims to discover novel genetic markers and molecular pathways associated with immune dysregulation in AD by analyzing comprehensive genomic data, transcriptomic profiles, and immune cell signatures. Additionally, the study intends to develop a robust diagnostic model capable of accurately differentiating AD patients from healthy individuals based on immune cell-related biomarkers.

## Materials and methods

### Data collection and processing

Three human AD microarray datasets, namely GSE85426, GSE63060, and GSE63061 were selected and downloaded from the GEO database (https://www.ncbi.nlm.nih.gov/geo/). The details of the selected datasets are presented in **Table 1**. The selection criteria were as follows: (i) the datasets include both AD and control samples, (ii) The sample type used is blood, and (iii) The total number of samples included in the analysis is not less than 150. After eliminating the batch effects by the “sva” package in R (11), GSE85426, GSE63060 and GSE63061 were merged and used as AD training cohort. In addition, the E-MTAB-6094 dataset, including blood samples from 22 AD patients and 13 controls, was downloaded from the Array Express database (https://www.ebi.ac.uk/arrayexpress/) and used as an AD testing cohort. The “Limma” package (12) in R was used to identify DEGs between AD and control samples with FDR < 0.05 and |log_2_FC| > 0.5 as the cutoff threshold. The results were visualized in a volcanic plot and heatmap.

**Table 1:** The information of selected datasets in this study.

ID	Platform	Total sample number	CTRL	AD
GSE85426	GPL14550 Agilent	180	90	90
GSE63060	GPL6947 Illumina	249	104	145
GSE63061	GPL10558 Illumina	273	134	139

### Evaluation of immune cell infiltration and their relationship with DEGs

The CIBERSORT algorithm was applied to calculate the proportion of 22 immune cells based on gene expression profiles of merging datasets. Student-t test was used to identify differentially infiltrated immune cells (DIICs) between AD and control samples in the training cohort with p < 0.05 as the cutoff threshold. Next, the Cor function of R was applied to perform correlation analysis between DIICs and DEGs to screen DIIC-correlated DEGs with p < 0.05 and |cor| > 0.3 as the cutoff threshold. The function of DIIC-correlated DEGs was analyzed by DAVID (13, 14), and GO terms and KEGG pathways with FDR < 0.05 were considered significantly enriched. In addition, the interactions among DIIC-correlated DEGs were evaluated in the STRING database (https://string-db.org/), followed by the construction of PPI network using DIIC-correlated DEGs with interaction score greater than 0.6 by Cytoscape software (15).

### WGCNA analysis

WGCNA was carried out to build modules related to DIICs based on the gene expression profiles (16). After calculating the Pearson correlation coefficient, a similarity matrix was constructed. Then the similarity matrix was converted into an adjacency matrix, in which the optimal soft threshold was selected to build a scale-free network. Then adjacency matrix was converted to TOM for hierarchical clustering analysis of genes, followed by the identification of network modules by Dynamic TreeCut setting cut height as 0.995. Next, the correlations between modules and DIICs were calculated. To further narrow the scope of DIIC-correlated DEGs, Fisher’s exact test was performed by projecting DIIC-correlated DEGs into each module. DIIC-correlated DEGs in modules with p < 0.05 and FC >1 were selected for the following analysis.

### Identification of robust DIIC-related biomarkers by machine learning

Three machine learning methods, including LASSO, RFE, and RF, were applied independently to screen DIIC-related biomarkers. Briefly, LASSO was performed by “lars” package in R (17) to screen the gene signatures under the optimal lambda with the slightest classification error. RF was performed by “randomForest” package in R (18), and gene features and their contributions were then analyzed by explaining the function of the “DALEX” R package (19, 20); SVM was performed by “caret” package in R at 100-fold cross-validation to determine the variables at the max accuracy (20). At last, the overlapped genes identified by LASSO, RF, and SVM were considered robust DIIC-related biomarkers in AD (21, 22). The expressions of DIIC-related biomarkers in AD and control samples from training and testing cohorts were compared and displayed in the bar charts.

### Construction and validation of a diagnostic nomogram in AD

Then the overlapped genes were included in multivariate Cox regression analysis to construct the nomogram in training set by the “rms” package of R (23). The decision curves were plotted by the “rmda” package (24) of R to evaluate the clinical utility of the nomogram (25). In addition, the nomogram was tested in testing set. Furthermore, chemicals associated with AD and those biomarkers were searched in the CTD database (http://ctdbase.org/) and were employed to construct a chemical-gene network by Cytoscape software.

### Quantitative real-time reverse transcriptions PCR

Total RNA was extracted from the peripheral blood of six patients with Alzheimer’s disease and six healthy adults from Liaocheng Hospital of Shandong First Medical University using Trizol reagent (AG21101, AG, CHINA). Inclusion criteria: people who met the diagnostic criteria for AD (NIA-AA criteria) published by the National Institute on Aging (NIA) and the Alzheimer’s Association (AA) in 2011 (Alzheimer’s disease group); people without cognitive impairment (control group).

Exclusion criteria: Patients with a clear history of stroke; Patients with psychiatric disorders; History of the immune system and severe hematologic disorders. Informed consent was obtained from the subjects or their legal guardians, and the Ethics Committee of Liaocheng Hospital, Shandong First Medical University, approved the study (NO.2023039).

RNA was reverse transcribed into cDNA with a commercial reverse transcription kit (AG11706, AG, CHINA), and quantitative real-time PCR was performed by the SYBR premixed Ex Taq kit (AG11718, AG, CHINA) and specific primers (**Table 2**). Glyceraldehyde 3-phosphate dehydrogenase (GAPDH) was used as the reference gene. Relative mRNA levels were expressed and calculated using the 2(-Delta CT) method. then subjected to one-way analysis of variance using Graphpad Prism8 software, and *P* value < 0.05 was considered a significant difference.

**Table 2:** Primers used for RT-PCR in this study.

Gene	Forward primer(5′-3′)	Reverse primer(5′-3′)
CMTM2-F	AAGAAGGACGGTAAGGAGCCA	GCACCGCCTTTTGAGGTTTG
DDIT4-F	TGGGCAAAGAACTACTGCG	AGAGTTGGCGGAGCTAAACAG
LDHB-F	CCTCAGATCGTCAAGTACAGTCC	ATCACGCGGTGTTTGGGTAAT
NDUFA1-F	ATGTGGTTCGAGATTCTCCCC	CCTGTGGATGTACGCAGTAGC
NDUFS5-F	TGCACATGGAATCGGTTATACTC	CCGAAGCAAACACTCTACGAAAT
RPL17-F	GAACACTCGTGAAACTGCTCA	AACGTCGGAATGGTACACACT
NDUFB2-F	GGAGGCCGCCTTTTCAGAA	GGAAGGATCAGGATACGGAAAGT
RPL21-F	CAAGGGAATGGGTACTGTTCAAA	CTCGGCTCTTAGAGTGCTTAATG
RPL26-F	GACTTCCGACCGAAGCAAGAA	TGCACCCGTTCAATGTAGATAAC
NDUFAF2-F	ATGCCTCTGCTCCATACTTTGG	TGGCTCTTGCCATCTCGTG

## Results

### Identification of DEGs in AD training cohort

**Figure 1:**
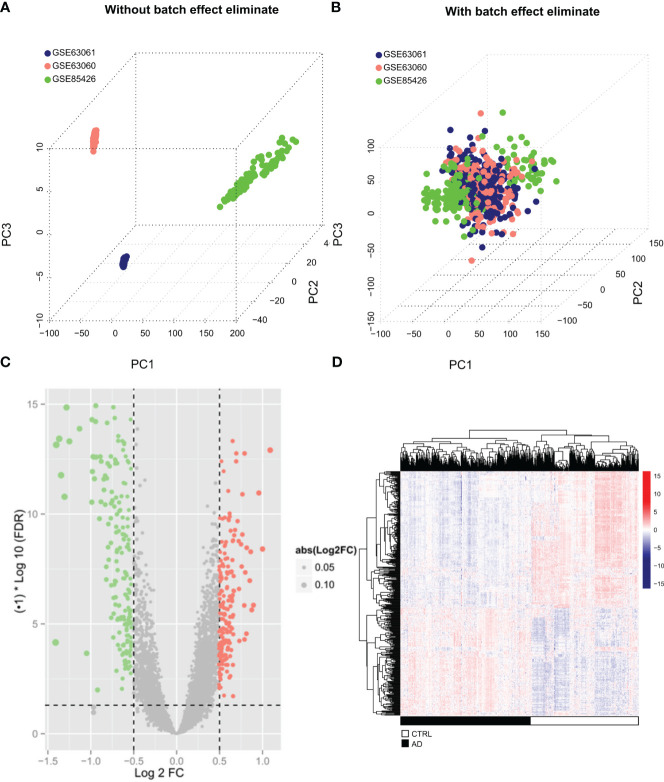
Screening of significantly differentially expressed genes **(A, B)** The three data sets (GSE85426, GSE63060, GSE63061) remove the batch effect through the “sva” algorithm and merge them into one data set. The sample relationship before and after the batch effect removal. **(C)** The volcano map of DEGs. The green and red dots represent significantly lower and higher DEGs, respectively, the black horizontal line represents FDR<0.05, and the two vertical lines representlog2FC |>0.5. **(D)** Heatmap of DEGs. Black and white in the sample bar represent the AD and the control groups, respectively.

Batch effects among GSE85426, GSE63060, and GSE63061 (**Figure 1A**) were first eliminated (**Figure 1B**) by “sva” package in R. A total of 404 DEGs, including 234 down-regulated and 170 up-regulated genes, were identified between AD (N = 374) and control (N = 328) samples in the merging AD training datasets (**Table S1**) and displayed in the volcanic plot (**Figure 1C**) and heatmap (**Figure 1D**).

### Identification of DEGs correlated with key infiltrated immune cells in AD

**Figure 2:**
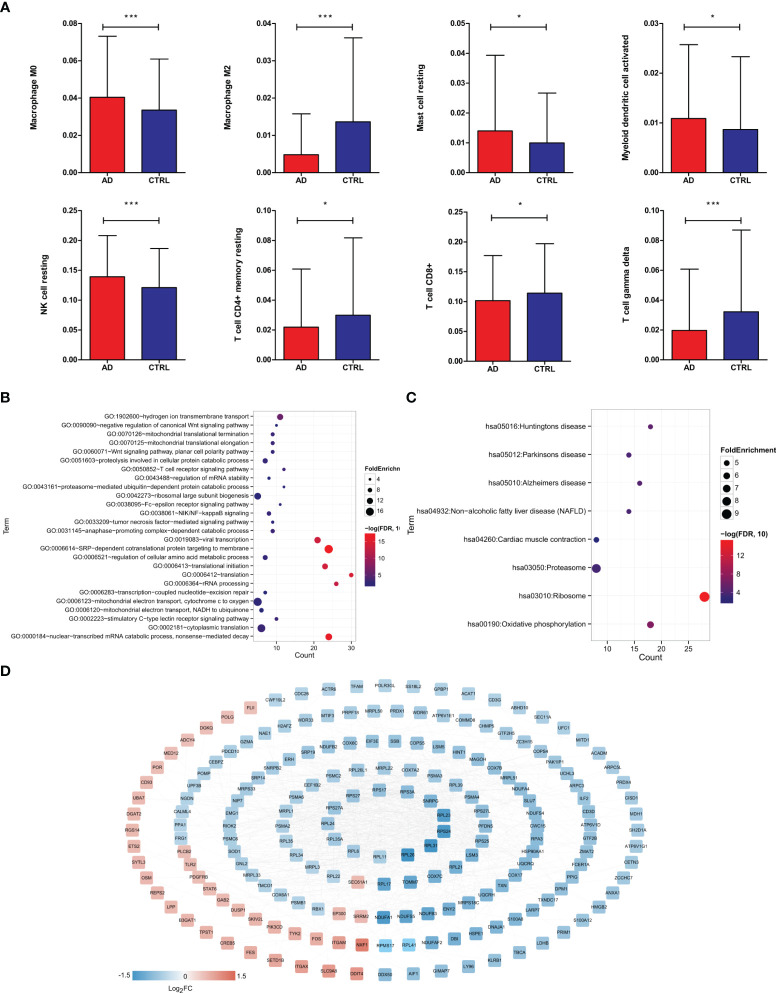
Screening of DEGs significantly related to immune cells. **(A)** Distribution histogram of immune cells with significantly different distribution in different groups based on CIBERSORT evaluation. Immunologically significantly correlated DEGs, significantly correlated GO biological process, *P < 0.05, ***P < 0.001. **(B)** and KEGG signal pathway **(C)** bubble diagram, the horizontal axis represents the number of genes, the vertical axis represents the item name, the size of the dot represents the FoldEnrichment value, and the color represents significance. The larger the dot, the closer the color is and the higher the significance. **(D)** Interaction network diagram, color indicates the degree of significant difference.

To identify key immune cells in AD, CIBERSORT was carried out to calculate the proportions of 22 immune cells in AD and control samples (**Table S2**). We found that compared to control samples, AD patients had significantly higher proportions of resting NK cells, M0 macrophages, activated myeloid dendritic cells, resting mast cells and lower proportions of CD8+ T cells, resting memory CD4+ T cells, gamma delta T cells, and M2 macrophages (**Figure 2A**), suggesting that those DIICs were key immune cells in AD. Next, 277 DEGs were detected to be significantly correlated with those DIICs (**Table S3**). Functional analysis showed that DIICs-correlated DEGs were significantly enriched into 26 GO terms (**Figure 2B**), such as SRP-dependent cotranslational protein targeting to membrane, nuclear-transcribed mRNA catabolic process, nonsense-mediated decay, translation, and 8 KEGG pathways (**Figure 2C**), including ribosome, oxidative phosphorylation, Huntington’s disease, Alzheimer’s disease, Parkinson’s disease, non-alcoholic fatty liver disease (NAFLD), proteasome and cardiac muscle contraction. Moreover, the PPI network, including 202 nodes and 1,096 interaction pairs, revealed close interactions among DIIC-correlated DEGs (**Figure 2D**).

### Further screening of DIIC-correlated DEGs by WGCNA

**Figure 3:**
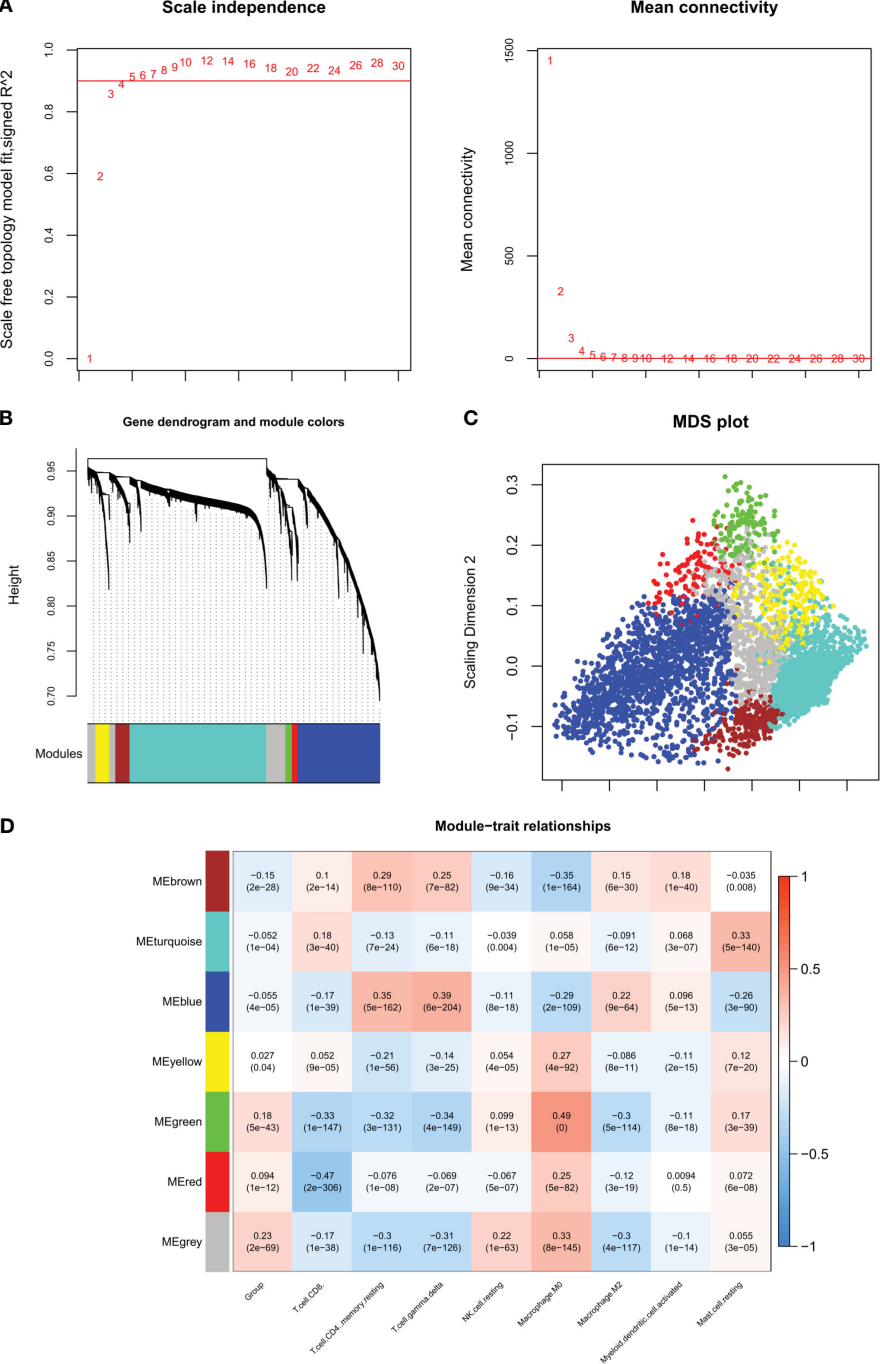
WGCNA algorithm screening disease progression and immune-related modules. **(A)** Left figure: The weight parameter power selection diagram of the adjacency matrix. The horizontal axis represents the weight parameter power, and the vertical axis represents the square of the correlation coefficient between log (k) and log (p (k)) in the corresponding network. The red line represents the standard line where the square value of the correlation coefficient reaches 0.9. Right figure: Schematic diagram of average connectivity of genes under different power parameters. The red line indicates that under the weight parameter power of the adjacency matrix in the left figure, the average connectivity of network nodes is 1. **(B)** Gene dendrogram and module colors, each color represents a different module. **(C)** MDS diagram of DEGs contained in each module. **(D)** Correlation heat map between the proportion of sample immune cells and the partition of each module.

A total of 277 DIIC-related DEGs were analyzed in WGCNA, the soft threshold power five was chosen to construct a scale-free network at R^2 = ^0.9 (**Figure 3A**). Then 7 modules were identified by Dynamic TreeCut (**Figure 3B**), and the multidimensional scaling analysis showed that genes in the same module were distributed in the same region (**Figure 3C**). Next, DIIC-correlated DEGs were projected to those modules. Fisher’s exact test revealed that DIIC-correlated DEGs were significantly enriched in blue, brown, green, and red modules (**Table 3**). The module-trait correlation analysis showed that the blue module was positively correlated with resting memory CD4 T cells (cor = 0.35) and delta gamma T cells (cor = 0.39), the brown module was negatively correlated with M0 macrophages (cor = -0.35), the green module was positively correlated with M0 macrophages (cor = 0.49) and negatively correlated with CD8 T cells (cor = -0.33), resting memory CD4 T cells (cor = -0.32), delta gamma T cells (cor = -0.34) and M2 macrophages (cor = -0.3), red module was negatively correlated with CD8 T cells (cor = -0.47) (**Figure 3D**). Thus, 118 DIIC-correlated DEGs in the blue module, 17 DIIC-correlated DEGs in brown module, 30 DIIC-correlated DEGs in green module, and 12 DIIC-correlated DEGs in the red module were used for the downstream analysis (**Table S4**).

**Table 3:** Fisher’s exact test to identify key modules.

ID	Color	Module size	DIIC correlated DEGs	Enrichment fold[95%CI]	Phypers
module 1	blue	1573	118	2.229[1.7439-2.839]	1.35E-10
module 2	brown	276	17	1.830[1.029-3.063]	2.90E-02
module 3	green	123	30	7.243[4.569-11.19]	1.55E-14
module 4	grey	652	8	0.365[0.154-0.737]	2.54E-03
module 5	red	116	12	3.073[1.516-5.701]	1.24E-03
module 6	turquoise	2643	3	0.033[0.00688-0.100]	2.20E-16
module 7	yellow	263	2	0.226[0.0270-0.836]	1.77E-02

### Construction and validation of a DIIC-related diagnostic model in AD

**Figure 4:**
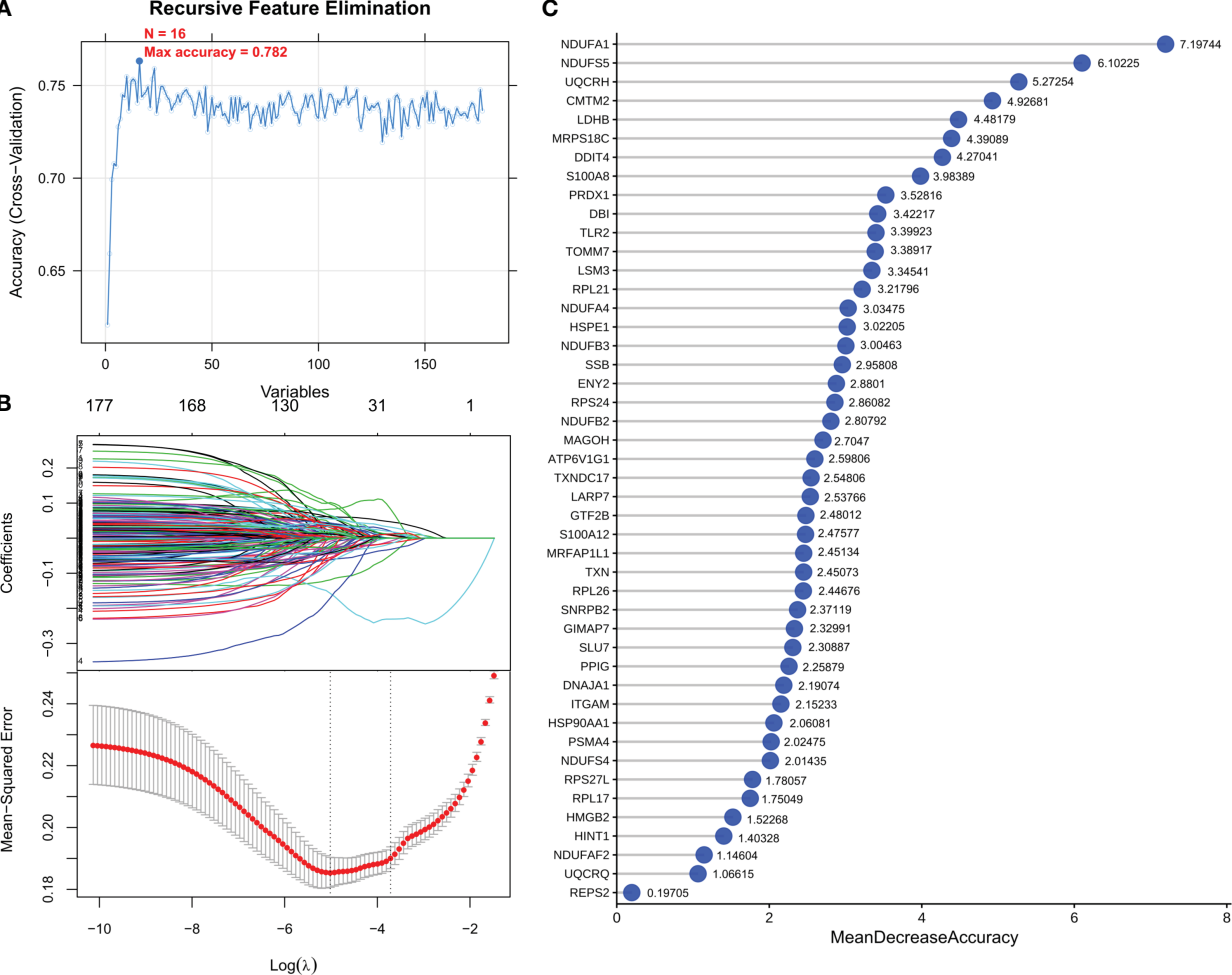
Optimization algorithm for screening important immune-related markers. DEGs parameter diagram of RFE **(A)**, LASSO algorithm **(B)** and RF **(C)** filtering features.

**Figure 5:**
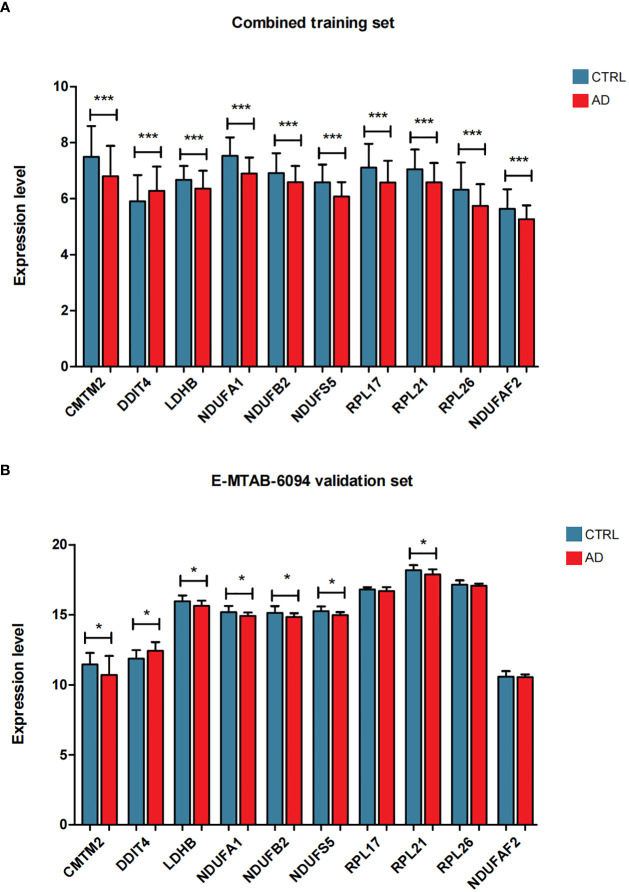
The expression level distribution of characteristic genes in the combined training data set **(A)** and E-MTAB-6094 validation data set **(B)**, *P < 0.05, ***P < 0.001.

**Figure 6:**
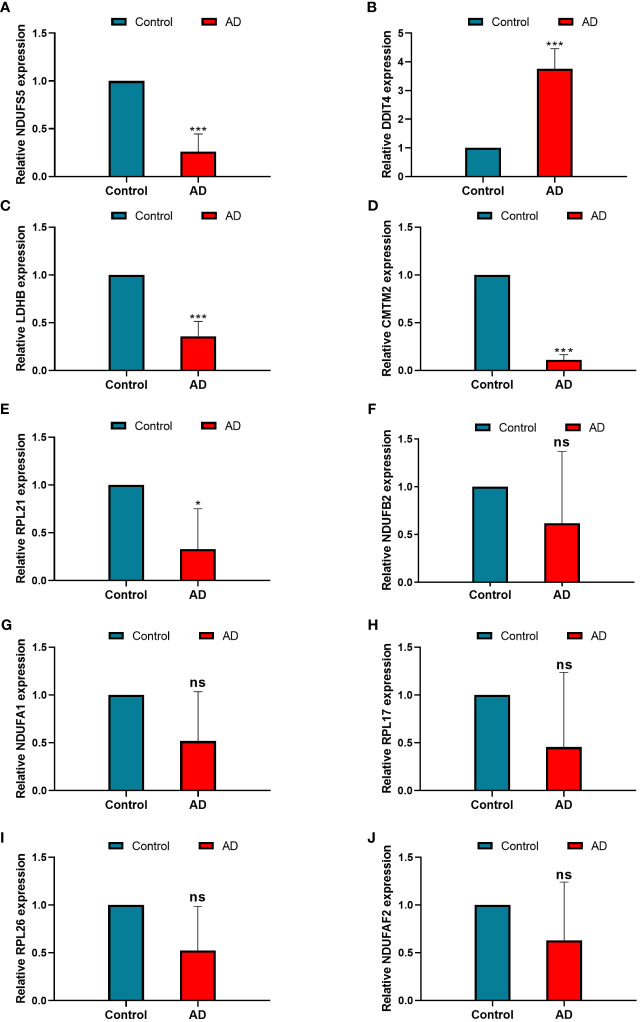
The relative expression levels of NDUFS5 **(A)**, DDIT4 **(B)**, LDHB**(C)**, CMTM2 **(D)**, RPL21 **(E)**, NDUFB2 **(F)**, NDUFA1 **(G)**, RPL17 **(H)**, RPL26 **(I)**, and NDUFAF2 **(J)** in the model between control and AD samples identified by RT-PCR, GAPDH was used as a reference. * *P* < 0.05, ***P < 0.001, ns, non-significant.

**Figure 7:**
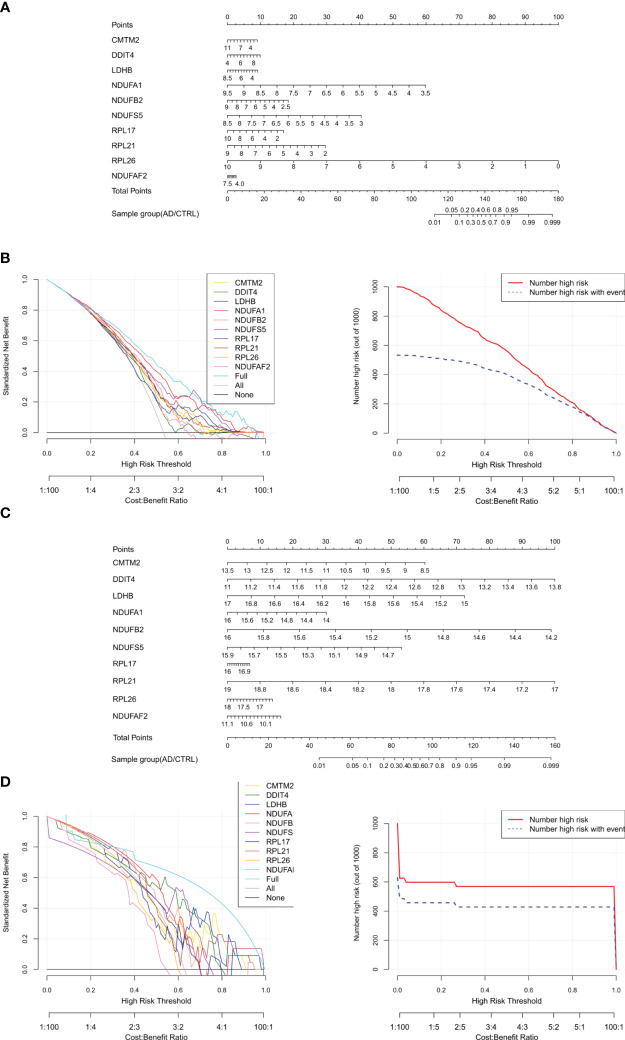
Construction and verification of Nomogram diagnostic model. **(A)** Nomogram model diagram based on the expression level of immune DEGs in the combined training data set based on ten features. **(B)** Model decision line diagram. **(C)** Nomogram model diagram of the expression level of immune DEGs in E-MTAB-6094 validation data set based on ten characteristics. **(D)** Model decision line diagram.

**Figure 8:**
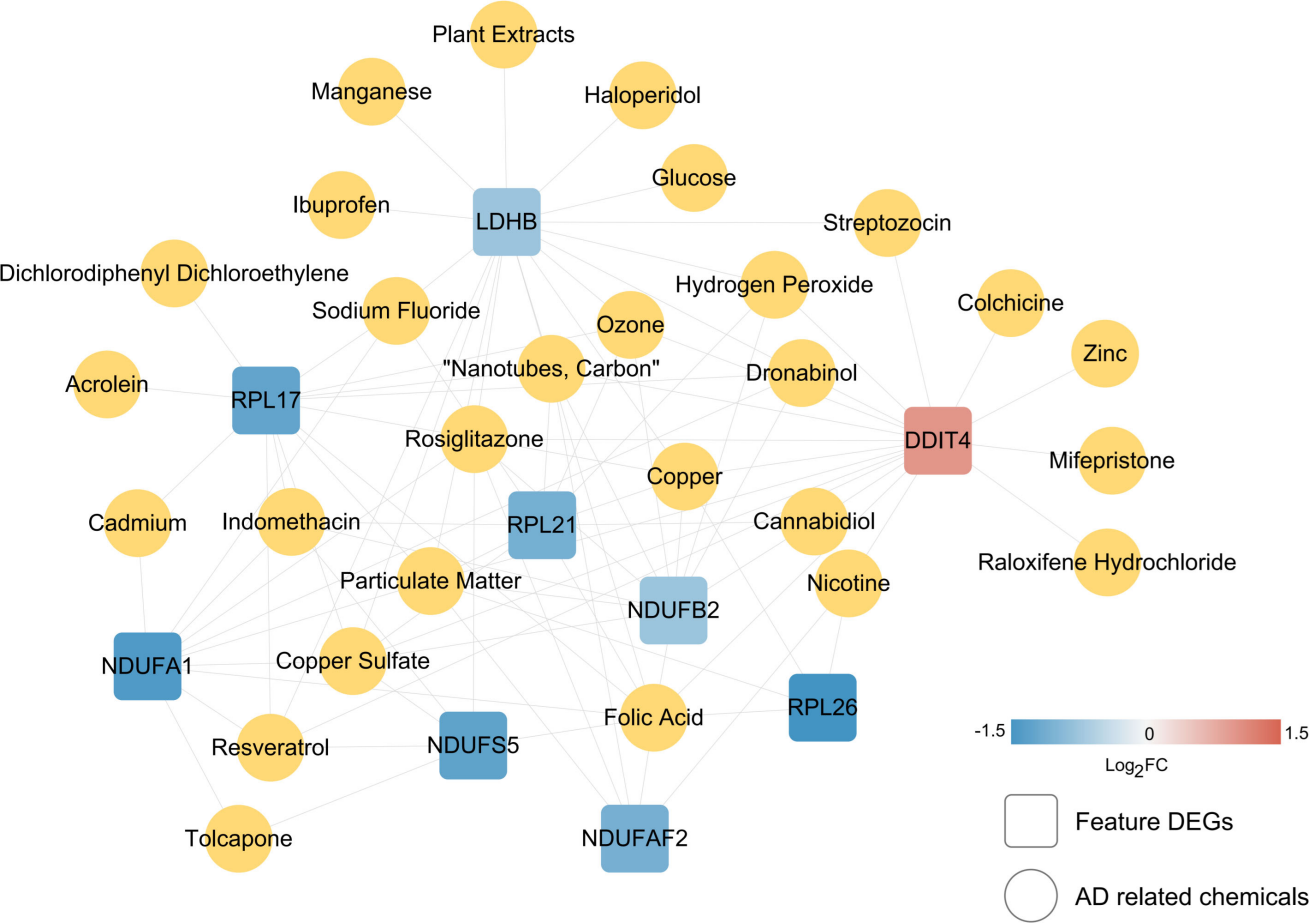
Small molecule screening of chemical drugs related to characteristic immune DEGs. The linkage map of 10 characteristic genes and small chemical molecules. The square and yellow circles represent small molecules of characteristic genes and AD-related chemicals.

To screen robust DIIC-related biomarkers in AD, different machine-learning methods were employed to screen robust DIIC-related biomarkers in AD. Using RFE, 16 genes were identified as potential biomarkers for AD (**Table S5**, **Figure 4A**). By LASSO, 37 signatures were extracted as candidate biomarkers (**Table S6**, **Figure 4B**). 46 genes were identified by the RF algorithm (**Table S5** and **Figure 4C**). At last, 10 overlapping genes, including CMTM2, DDIT4, LDHB, NDUFA1, NDUFB2, NDUFS5, RPL17, RPL21, RPL26 and NDUFAF2, were obtained and considered as robust biomarkers in AD. Except for DDIT4, the expressions of the other nine genes significantly decreased in AD compared with those in controls in the training set (**Table S6**, **Figure 5A**). Furthermore, the expression trend of these ten genes is similar in the test set, with significant differences in gene CMTM2, DDIT4, LDHB, NDUFS5, and RPL21 (**Table S6**, **Figure 5B**). Six Alzheimer patients and six healthy controls were included in the study (**Table 4**), and the expression trends of these genes verified by peripheral blood qPCR were consistent with the results of our previous bioinformatics analysis (**Figure 6**). Based on CMTM2, DDIT4, LDHB, NDUFA1, NDUFB2, NDUFS5, RPL17, RPL21, RPL26 and NDUFAF2, a nomogram was constructed (**Figure 7A**). The decision curves showed that the patients got the highest benefits from the combined nomogram model compared with the other single biomarker models (**Figure 7B**). The nomogram was also constructed in the testing cohort (**Figure 7C**), and the decision curves in the testing cohort further demonstrated the efficient clinical utility of the combined nomogram model (**Figure 7D**). Moreover, candidate chemicals targeting those biomarkers in AD were screened in the CTD database; a total of 28 chemicals, such as 4-hydroxy-2-nonenal, rosiglitazone, resveratrol, cannabidiol, colchicine were identified, followed by the construction of chemical-biomarker network composed of 94 chemical-biomarker pairs (**Figure 8**, **Table S7**).

**Table 4:** Sample details.

Age (years)	Sex	Diagnosis	Sample Collection
75	male	Alzheimer’s disease	Peripheral blood
67	male	Alzheimer’s disease	Peripheral blood
86	female	Alzheimer’s disease	Peripheral blood
79	female	Alzheimer’s disease	Peripheral blood
74	female	Alzheimer’s disease	Peripheral blood
77	female	Alzheimer’s disease	Peripheral blood
60	male	Health controls	Peripheral blood
70	female	Health controls	Peripheral blood
68	female	Health controls	Peripheral blood
77	male	Health controls	Peripheral blood
82	male	Health controls	Peripheral blood
73	female	Health controls	Peripheral blood

## Discussion

Neuroimmune refers to the intricate interplay between the nervous and immune systems, encompassing the coordinated regulation of diverse cell types, including neurons, glial cells, and immune cells (26). Recent research has shed light on the pivotal role of aberrant activation of neuroimmune cells and heightened inflammatory responses in the progression of Alzheimer’s disease (AD) (10). This dysregulated activation of immune cells and the ensuing surge in inflammatory reactions lead to irreversible damage to neurons and the delicate functioning of the nervous system (10). However, the diagnostic value of immune cell-related genes in AD remains unclear. In the current study, a comprehensive analysis by CIBERSORT, WGCNA, and machine learning identified 10 immune cell-related genes in blood samples, followed by the construction of a reliable diagnostic nomogram and candidate chemicals in AD treatment. We hope to improve our understanding of the relationship between immune cells and AD at a genetic level and provide useful information for the treatment of AD patients.

The trends of expression levels of the ten hub genes obtained from this screening were identical in the training and validation sets, and to further validate the accuracy of our bioinformatics analysis, we verified their expression differences in peripheral blood in AD patients and healthy populations and obtained consistent results. Furthermore, evidence suggests that immune cell populations promise as diagnostic and prognostic biomarkers for AD (27). For example, a recent study demonstrated significant differences in the ratios of CD4+ T cells, NK cells, CD8+ T cells, and monocyte-macrophages between AD patients and healthy individuals at the single-cell level (28). These findings indicate that immune cell alterations and dysregulation may indicate disease status and progression.

CMTM2 is a member of a newly found gene family (chemokine-like factor-like MARVEL transmembrane domain-containing family members), the members which have been reported to be involved in various tumors, reproduction, and immunity (29). CMTM2 has been reported in spermiogenesis (30), hepatocellular carcinoma, HBV-related disorders (31), and gastric cancer (32). However, its role in immunity and the nervous system is unknown. In the current study, we found that the expression of CMTM2 significantly decreased in AD and was significantly correlated with M2 macrophages and gamma delta T cells (**Table S3**), which were DIICs between AD and controls. In AD, decreased M2 macrophage subset has been reported to indicate worse cognitive performance (33). Transplanting of M2 macrophages could reduce neuron loss, impair inflammation response and enhance cognitive ability (34). Gamma delta T cells produce IL-17, the accumulation of which was shown to be concomitated with the onset of cognitive decline in female AD mice model (35). Therefore, CMTM2 may regulate AD via M2 macrophages and gamma delta T cells, which needs further study.

The encoded product of DDIT4, RTP801 (also known as REDD1), is involved in Aβ-induced synaptic dysfunction by regulating Aβ’s cytotoxicity (36, 37), and knockdown of DDIT4 could improve cognitive ability and ameliorate neuroinflammation severity (38, 39). Those findings indicate that DDIT4 may be a promising therapeutic target in AD. In addition, our study found that the expression of DIIT4 was significantly correlated with resting NK cells. Consistent with our study, the proportion of resting NK cells was also significantly different between AD and control samples in other studies (40, 41), indicating an essential role of resting NK cells in AD. It has been reported that immune infiltration of NK cells in the brain may contribute to neuroinflammation in AD (42). However, the specific role of resting NK cells in the blood remains unclear. Given studies on DIIT4, it is inferred whether the role of DDIT4 in neuroinflammation is associated with resting NK cells.

In our study, the expression of LDHB was significantly decreased in AD samples and was correlated with resting mast cells and resting memory CD4+ T cells. LDHB is a vital player in lactate metabolism (43). Alteration of lactate metabolism was observed to have a close relationship with neuronal damage in AD mice model (44). In tumors, overexpression of LDHB could mitigate the effects of lactic acid on CD4 T cell-mediated cytokine production (45). However, to our knowledge, the role of LDHB on the CD4 T cells’ function in AD is unknown. Mast cells are involved in neuroprotection and neuroinflammation by secreting inflammatory mediators and cytokines (46), and activation of mast cells may accelerate the development of AD (47). Thus, LDHB may be involved in AD via lactate metabolism and neuroinflammation mediated by resting CD4+ T cells and resting memory cells.

RPL17, RPL21, and RPL26 are all ribosomal proteins. Among them, RPL26 was reported to be involved in methylation within AD neurons (48). However, the exact role of RPL26 in AD remains unclear. In the current study, we found that RPL17, RPL21, and RPL26 expressions correlated with M2 macrophages and gamma delta T cells. It will be, therefore, of interest in future studies to test the relationship between them and their role in modulating cognitive ability in AD.

NDUFA1, NDUFB2, NDUFS5, and NDUFAF2 are ubiquinone oxidoreductase subunits or complex assembly factors which play an important role in oxidative stress (49–51). Oxidative stress may be essential to AD development by promoting Aβ deposition, tau hyperphosphorylation, and the subsequent loss of synapses and neurons (52). However, the studies on those genes in AD are limited. Gly32Arg SNP is a mutational site in NDUFA1 Reported that may be associated with early-onset dementia (53). Interestingly, we found that those genes are also significantly correlated with M2 macrophages and gamma delta T cells, further indicating the critical role of M2 macrophages and gamma delta T cells in AD.

AD lacks effective treatment measures in clinical practice (54); Immunotherapy has garnered considerable attention as a promising treatment strategy aimed at modulating immune system function, reducing inflammation, and clearing pathological protein deposits to improve the disease condition and alleviate symptoms (55).

This study utilized gene expression profiling and bioinformatics methods to identify abnormal expression patterns of immune cell-related genes in individuals with Alzheimer’s disease (AD). Based on the these Hub genes, an effective diagnostic model of Nomogram was constructed and chemical drugs targeting these genes were predicted for the treatment of AD.These findings potentially reflect alterations within the immune system, including immune cell activation, cellular infiltration levels, and inflammatory responses. Investigating the mechanisms underlying immune cell activation, dysfunction, and interplay, as well as the regulatory networks of immune genes, holds promise for unraveling the pathogenesis of AD and establishing a theoretical foundation for developing novel therapeutic strategies. Nevertheless, certain limitations should be acknowledged in our current study. Firstly, although PCR validation confirmed the expression of immune cell-related biomarkers in the peripheral blood of AD patients and healthy volunteers, further comprehensive investigations are required to elucidate the specific mechanisms underlying the actions of these genes. Secondly, understanding the relationship between immune cells, biomarkers, and their interactions in regulating the development of AD necessitates further exploration.

## Conclusion

In summary, our study identified CMTM2, DDIT4, LDHB, NDUFA1, NDUFB2, NDUFS5, RPL17, RPL21, RPL26, and NDUFAF2 as immune cell-related biomarkers and constructed a diagnostic model in AD. Our findings may provide novel ideas for managing and treating AD patients.

## Data availability statement

The datasets in this study can be found in online repositories. The names of the repository/repositories and accession number(s) can be found below: https://www.ncbi.nlm.nih.gov/, GSE85426, GSE63060 and GSE63061. https://www.ebi.ac.uk/arrayexpress/, E-MTAB-6094.

## Ethics statement

The studies involving human participants were reviewed and approved by the Institutional Ethical Committee of the Liaocheng People’s Hospital and Liaocheng Hospital Affiliated to Shandong First Medical University (NO.2023039). The patients/participants provided their written informed consent to participate in this study.

## Author contributions

XZ, MB, and GJ conceived and drafted the article, GZ and ZW provided bioinformatics analysis, GZ and HW provided samples and conducted RT-PCR test. All authors contributed to the article and approved the submitted version.
